# Supplementary material for: Splice-Junction-Based Mapping of Alternative Isoforms in the Human Proteome
Source: Cell Rep. Author manuscript; Available in PMC 2020 Jan 15. (PMC6961840; doi:10.1016/j.celrep.2019.11.026)
Supplement: 2 [file NIHMS1546469-supplement-2.zip › DF1/PXD006675/SmoothMuscleCells/SmoothMuscleCells_6_SRRM1_KVELSESEDK.pdf]

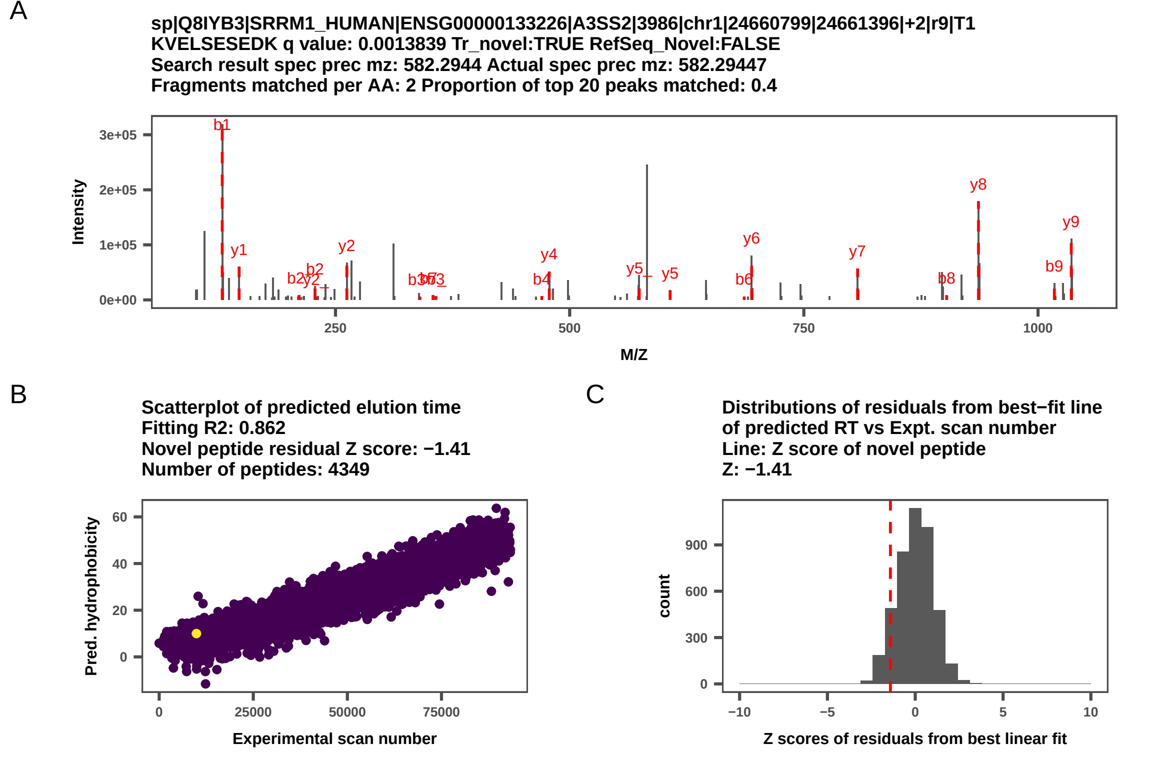
A
sp|Q8IYB3|SRRM1_HUMAN|ENSG00000133226|A3SS2|3986|chr1|24660799|24661396|+2|r9|T1
KVELSESEDK q value: 0.0013839 Tr_novel:TRUE RefSeq_Novel:FALSE
Search result spec prec mz: 582.2944 Actual spec prec mz: 582.29447
Fragments matched per AA: 2 Proportion of top 20 peaks matched: 0.4
Intensity
M/Z
B
Scatterplot of predicted elution time
Fitting R2: 0.862
Novel peptide residual Z score: −1.41
Number of peptides: 4349
Pred. hydrophobicity
Experimental scan number
C
Distributions of residuals from best−fit line
of predicted RT vs Expt. scan number
Line: Z score of novel peptide
Z: −1.41
count
Z scores of residuals from best linear fit
